# Supplementary material for: The Construction and Exploration of a Comprehensive MicroRNA Centered Regulatory Network in Foxtail Millet (Setaria italica L.)
Source: Front Plant Sci. 2022 May 6;13:848474. doi: 10.3389/fpls.2022.848474 (PMC9121102; doi:10.3389/fpls.2022.848474)
Supplement: Supplementary file 7 [file Data_Sheet_7.zip › Sit-miR408_Seita.1G363800.1_366_TPlot.pdf]

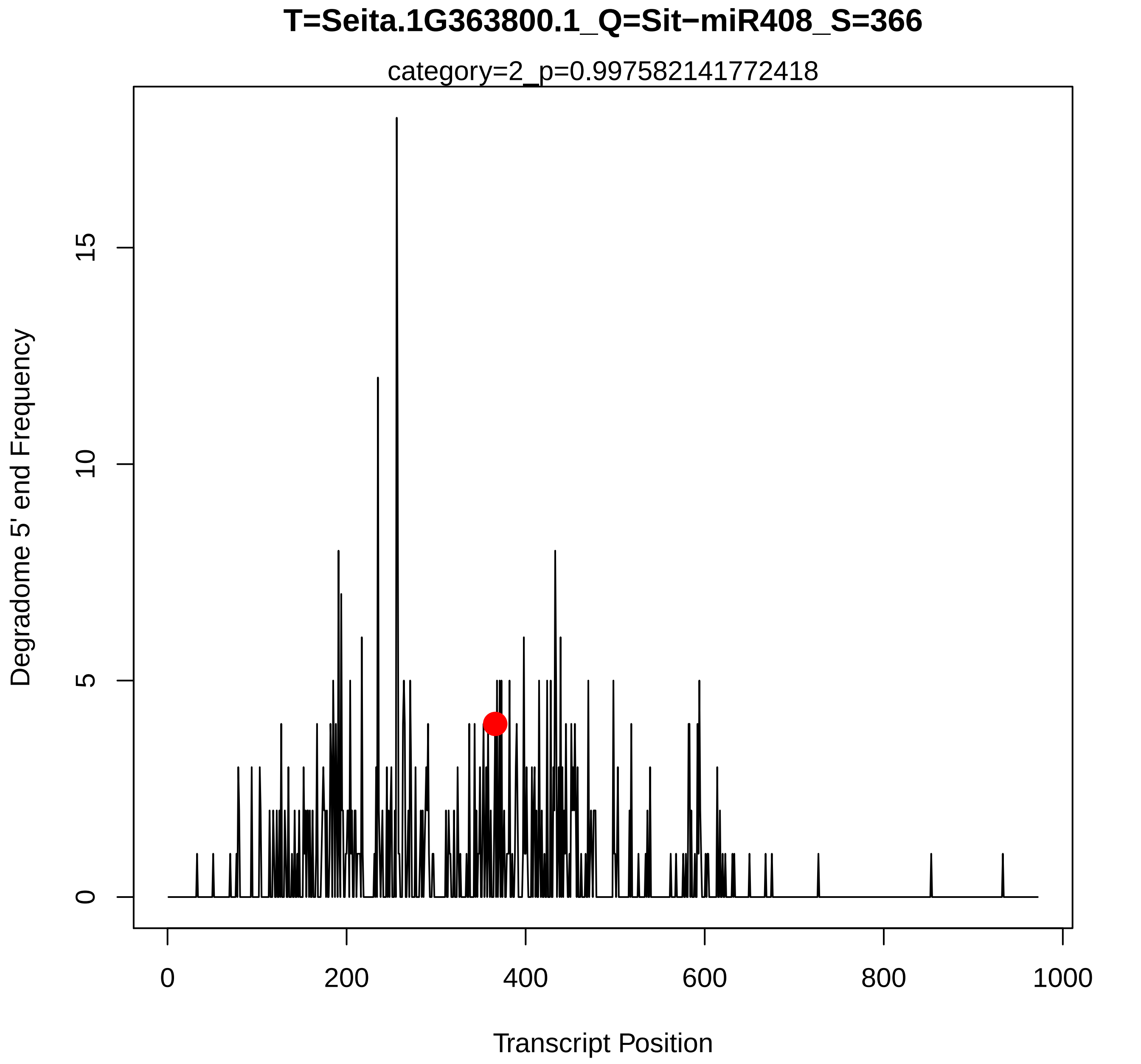
T=Seita.1G363800.1_Q=Sit−miR408_S=366
category=2_p=0.997582141772418
Degradome 5' end Frequency
0
5
10
15
Transcript Position
0
200
400
600
800
1000
